# Effects of silica nanoparticle exposure on mitochondrial function during neuronal differentiation

**DOI:** 10.1186/s12951-017-0284-3

**Published:** 2017-07-04

**Authors:** Angélique D. Ducray, Andrea Felser, Jana Zielinski, Aniela Bittner, Julia V. Bürgi, Jean-Marc Nuoffer, Martin Frenz, Meike Mevissen

**Affiliations:** 1 Division of Pharmacology and Toxicology, Vetsuisse Faculty, University of Bern, Laenggassstrasse 124, 3012 Bern, Switzerland; 2 Institute of Clinical Chemistry, University Hospital Bern, 3010 Bern, Switzerland; 3 Institute of Applied Physics, University of Bern, Sidlerstrasse 5, 3012 Bern, Switzerland

**Keywords:** Nanomedicine, Silica-nanoparticle, Neuronal differentiation, Mitochondrial respiration

## Abstract

**Background:**

Nanomedicine offers a promising tool for therapies of brain diseases, but potential effects on neuronal health and neuronal differentiation need to be investigated to assess potential risks. The aim of this study was to investigate effects of silica-indocyanine green/poly (ε-caprolactone) nanoparticles (PCL-NPs) engineered for laser tissue soldering in the brain before and during differentiation of SH-SY5Y cells. Considering adaptations in mitochondrial homeostasis during neuronal differentiation, metabolic effects of PCL-NP exposure before and during neuronal differentiation were studied. In addition, kinases of the PI3 kinase (PI3-K/Akt) and the MAP kinase (MAP-K/ERK) pathways related to neuronal differentiation and mitochondrial function were investigated.

**Results:**

Differentiation resulted in a decrease in the cellular respiration rate and the extracellular acidification rate (ECAR). PCL-NP exposure impaired mitochondrial function depending on the time of exposure. The cellular respiration rate was significantly reduced compared to differentiated controls when PCL-NPs were given before differentiation. The shift in ECAR was less pronounced in PCL-NP exposure during differentiation. Differentiation and PCL-NP exposure had no effect on expression levels and the enzymatic activity of respiratory chain complexes. The activity of the glycolytic enzyme phosphofructokinase was significantly reduced after differentiation with the effect being more pronounced after PCL-NP exposure before differentiation. The increase in mitochondrial membrane potential observed after differentiation was not found in SH-SY5Y cells exposed to PCL-NPs before differentiation. The cellular adenosine triphosphate (ATP) production significantly dropped during differentiation, and this effect was independent of the PCL-NP exposure. Differentiation and nanoparticle exposure had no effect on superoxide levels at the endpoint of the experiments. A slight decrease in the expression of the neuronal differentiation markers was found after PCL-NP exposure, but no morphological variation was observed.

**Conclusions:**

PCL-NP exposure affects mitochondrial function depending on the time of exposure before and during neuronal differentiation. PCL-NP exposure during differentiation was associated with impaired mitochondrial function, which may affect differentiation. Considering the importance of adaptations in cellular respiration for neuronal differentiation and function, further studies are needed to unravel the underlying mechanisms and consequences to assess the possible risks including neurodegeneration.

## Background

Nanomedicine offers promising possibilities for therapy of brain diseases as drug carriers, in tumor destruction and laser tissue soldering. In the latter application, silica nanoparticles embedded with bovine serum albumin in a biodegradable implant can be used for treatment of aneurysms in the brain [1, 2]. Despite several advantages compared to the conventional technique of suturing including speed, immediate water tightness, reduced tissue trauma, and faster wound healing, the nanoparticles may cause potential adverse effects once released from the biodegradable scaffold.

In previous studies, nanoparticle uptake and the underlying mechanisms and effects of these silica nanoparticles were studied in microglial cells, primary hippocampal cultures, neuron-like cells (SH-SY5Y) and organotypic brain slices [3–5]. Nanoparticle exposure did not result in increased cytotoxicity and apoptosis in microglial and neuron-like cell lines even though a transient depletion of glutathione was found indicating reactive oxygen species (ROS) formation [3]. Moreover, nanoparticles were demonstrated to be taken up by microglial cells in a time- and particle-dependent manner [4]; the uptake in primary hippocampal cultures was time- and concentration-dependent [5]. For all brain cells analyzed, no modulation of inflammatory cytokine secretion and autophagy was observed [4, 5], but neuronal differentiation markers including mitogen-activated protein kinase/extracellular signal-related kinase (MAP-K/ERK) 1/2 and phosphatidyl-inositol 3-kinase/serine/threonine specific protein kinase (PI3-K/Akt) kinases were shown to be downregulated after nanoparticle exposure [5].

Mitochondrial dynamics, trafficking, turnover, and biogenesis play key roles in regulating the functional health of neurons. Mitochondria do not only support the energy demands of neuronal electrophysiology, but also mediate calcium homeostasis, integration of cell death/survival signals, and fatty acid metabolism [6]. The limited glycolytic potential and uncontrolled mitophagy were demonstrated to be linked to neurodegeneration with the protein kinases ERK1/2 and PTEN-induced kinase 1 (PINK1) being involved [6]. Not surprisingly, perturbations in mitochondrial function have long been centrally implicated in the pathogenesis of Parkinson’s disease [7, 8].

Mitochondria play an important role in cell metabolism during neuronal differentiation because this process requires metabolic adaptations [9], and the PI3-K/AKT and ERK pathways were reported to be required for the differentiation of retinoic acid (RA)—induced neuroblastoma cell differentiation [10]. MAP-K/ERK 1/2 was reported to be important for regulating mitochondrial function [11–13] as well as PI3-K/Akt/mechanistic target of rapamycin (mTOR) being a regulator in glucose metabolism during neuronal differentiation [14]. Phosphorylation of c-Jun *N*-terminal kinase (JNK), ERK and p38 mitogen-activated kinase (p38) were found in primary astrocytes after exposure to zinc oxide (ZnO) nanoparticles [15]. Silver nanoparticles have demonstrated to induce impairment of mitochondrial oxidative phosphorylation [16] and exposure to titanium dioxide (TiO_2_) nanoparticles significantly impaired mitochondrial function in a concentration- and time-dependent manner in astrocytes [17]. Silica nanoparticles effectively inhibited vascular endothelial growth factor (VEGF)—induced angiogenesis in vitro and ERK 1/2 activation [18].

Unlike other cells, neurons show limited glycolytic potential, and both insufficient and excessive mitophagy have been linked to neurodegeneration. Mitochondrial dynamics is important for neurogenesis and neuronal differentiation [9, 19].

An increase in glucose metabolism was demonstrated during neuronal differentiation with PI3K/Akt/mTOR signaling being a critical regulator in neuronal energy metabolism [14]. Activated MAP-Ks were shown to phosphorylate various transcription factors resulting in regulation of cell proliferation, differentiation, inflammatory responses, oxidative stress caused by ROS and apoptosis [20]. Kinases have been demonstrated to be involved in neurite elongation (PI3-K/Akt), neuronal survival and in synaptic plasticity (MAP-K/ERK) of neurons [21, 22]. Activation of kinases such as Akt and ERK and an increase in neuronal differentiation was shown after exposure of SH-SY5Y cells to silver nanoparticles [23].

Given the importance of mitochondrial function in neuronal health, an interaction with nanoparticles may have detrimental consequences. Our aims were to investigate the effects of poly-(ε-caprolactone) (PCL) silica nanoparticles on the respiratory capacity of differentiating SH-SY5Y cells and to analyze the effect of nanoparticle exposure on the expression and the activation of the protein kinases Akt and MAP-K before and during neuronal differentiation.

## Methods

### Cell culture

SH-SY5Y cells were obtained from ATCC (Manassas, VA, USA), and culturing as well as differentiation was done with some adaptations as previously described [5]. Briefly, at day in vitro (DIV) 0, SH-SY5Y cells were seeded at a density of 1 × 10^7^ cells per T75 for Western samples, 8 × 10^4^ cells per well in 24-well plates (Techno Plastic Products AG (TPP) Trasadingen, Switzerland) and maintained at non-differentiated state for 24 h in Dulbecco’s Modified Eagle Medium (DMEM) GlutaMAX™ medium (Life Technologies, UK) sodium pyruvate [1 mM], l-glutamine [2 mM], penicillin/streptomycin ([1 unit/ml], Life Technologies, UK) at 37 °C in an atmosphere of 5% CO_2_.

For the first 3 days of differentiation, cells were exposed to the same medium with a reduced FBS concentration (5%) and supplemented with retinoic acid [10 μM] (RA, Sigma, St Louis, USA). For the last 3 days, SH-SY5Y cells were grown in DMEM with only 1% FBS with RA [10 μM]. PCL-NP exposure was performed before differentiation (NP DIFF) or during differentiation (DIFF NP DIFF) on DIV1 or DIV4, respectively. The cells were exposed to PCL-NPs in the same medium supplemented with 1% FBS for 24 h.

### Nanoparticle exposure

Nanoparticle synthesis and characterization as well as the chemical and physical properties of the PCL-NPs have been described previously [3, 4, 24]. Briefly, a core shell nanoparticle system was developed consisting of a defined silica-core of 80 nm with a hydrophobic PCL coating acting as carrier system for ICG. The ICG dye was used as an absorbing dye for the laser-soldering procedure. These nanoparticles incorporating rhodamine dye in the silica-core (silica-RITC)-PCL were used to study their uptake into cells. The designed nanoparticles were characterized by scanning electron microscopy, infrared spectroscopy, dynamic light scattering, thermogravimetric analysis, fluorescence measurements, and two-photon microscopy. Size, shape, ICG concentrations, zeta potential (−25.4 mV), surface charge, surface chemistry and photo stability were evaluated and published previously [24]. A stock solution of [2.6 × 10^11^ PCL-NPs/ml] was prepared using 0.0025% DMSO (Sigma, USA) in Dulbecco’s phosphate buffered saline (DPBS, Gibco, Life Technologies, UK). The stock solution was sonicated three times for 5 min with cooling steps in between to enable homogeneous nanoparticle suspension just before treatment of the cells. The final concentration of PCL-NPs [2.6 × 10^10^ PCL-NPs/ml] used for all experiments was obtained by dilution of the stock with culture medium containing 1% FBS as previously reported [4, 5].

### Microplate-based respirometry

The mitochondrial oxygen consumption rate (OCR), which is a key metric of aerobic mitochondrial function, and the extracellular acidification rate (ECAR), which approximates glycolytic activity, were analyzed simultaneously using a standard mitochondrial stress test paradigm on the Seahorse Bioscience XF-24 analyzer (Agilent Technologies, CA, USA). Cells were assayed at DIV8 for OCR and ECAR measurements following the manufacturer’s instructions (Agilent Technologies, CA, USA). For each group, five independent experiments were performed with five samples per experiment.

Before analysis, cells were washed three times with unbuffered assay media [DMEM (Sigma, Switzerland) diluted in water without phenol red supplemented with Glutamax (1X), sodium pyruvate [1 mM] and glutamine [2 mM], penicillin-streptomycin cocktail (1X) (Life Technologies, UK)] and incubated 1 h in a CO_2_-free incubator at 37 °C. After the initial measurement of basal OCR and ECAR, sequential exposures to modulators of mitochondrial activity were injected in the microtiter plate. First, the inhibitor of ATP synthase oligomycin [1 µM] was added to determine leak respiration induced through passive proton leakage across the mitochondrial inner membrane. Next, the uncoupler of mitochondrial oxidative phosphorylation carbonyl cyanide-4-(trifuoromethoxy) phenylhydrazone (FCCP, [0.125 µM]) was added to assess maximally stimulated uncoupled respiration. Finally, the complex (C)III inhibitor, antimycin A [1 µM] together with CI inhibitor rotenone [1 µM], an inhibitor of mitochondrial NADH dehydrogenase, were added to determine extramitochondrial respiration. Optimal concentrations of oligomycin, FCCP, antimycin A and rotenone were determined before. Basal respiration or acidification was calculated using the mean of the four OCR or ECAR measurements before the first injection. Leak respiration and maximal respiration were calculated as the mean of three OCR measurement cycles after oligomycin or FCCP injection, respectively. Maximal acidification was calculated as the mean of three ECAR measurement cycles after oligomycin injection. OCR data were corrected for non-mitochondrial oxygen consumption under rotenone and antimycin A. After each experiment, cell numbers in each well were measured using the CyQUANT kit (Molecular Probes, OR, USA). OCR (pmol/min) and ECAR (mpH/min) values were normalized to corresponding cell numbers.

### Enzymatic activities of respiratory chain complexes

SH-SY5Y cells were cultured as described above and harvested on DIV8. Briefly, cells were washed twice in cold HBSS, scraped and centrifuged at 4 °C at 2000 rpm for 5 min. Dried cell pellets were then frozen at −80 °C until used. For respiratory chain enzyme activity measurements, cells were mechanically homogenized and sonicated in buffer containing [25 mM] potassium phosphate (pH 7.2), [5 mM] MgCl_2_, and [2.5 mg/ml] BSA. Activity measurements of reduced nicotinamide adenine dinucleotide (NADH) coenzyme Q reductase complex (C)I, succinate dehydrogenase (CII), ubiquinol-cytochrome c reductase (CIII), cytochrome c oxidase (CIV), Mg-ATPase (CV), and citrate synthase were determined separately by spectrophotometry as previously described [25].

### Phosphofructokinase activity (PFK)

Phosphofructokinase measurements were done according to the manufacturer’s datasheet (Phosphofructokinase Activity Colorimetric Assay Kit, Sigma, Switzerland). SH-SY5Y cells were seeded in T25 flasks (Techno Plastic Products AG (TPP), Trasadingen, Switzerland) at a density of 3 × 10^6^ cells per flask and treated as previously described above. Briefly, the protein was extracted by scraping off cells in 1 mL HBSS and centrifuged at 13.200×*g* for 10 min. The buffer was removed and the pellet was dissolved in 200 µL cold PFK assay buffer and centrifuged at 13.200×*g* for 10 min. The pellet was solubilized in 60 µL cold PFK buffer and 50 µL were transferred to a 96- well plate and a master mix consisting of PFK Assay Buffer, PFK Enzyme Mix, PFK Developer, ATP and PFK substrate was added. The absorbance (450 nm) was measured using a Synergy H1 multi-mode reader. The data were compared with the NADH standard curve ranging from [2 to 10 nmol]/well and values were normalized to 1 mg protein. The protein amount of each sample was determined with Pierce™ 660 nm Protein Assay Reagent (Thermo Fisher Scientific, Switzerland).

### Mitochondrial membrane potential

The mitochondrial membrane potential (ΔΨ_m_) was determined using tetramethylrhodamine methyl ester (TMRM, Invitrogen, Thermo Fisher Scientific, Switzerland), a lipophilic cationic fluorescent probe which accumulates within mitochondria depending on their ΔΨ_m_. The measurements were performed according to the supplier’s instructions. SH-SY5Y cells were cultured as described above. Briefly, the provided Image-iT TMRM Reagent and TMRM were diluted to [100 nM] in 1% FBS/DMEM medium. At DIV8, the medium was replaced with the staining solution for 30 min at 37 °C. Successively, the cells were washed twice with HBSS and the signal was measured at an emission of 488 nm and an extinction of 570 nm with a Synergy H1 multi-mode reader. Consequently, the protein amount of each sample was determined with OPA as described. The values were normalized to 1 mg protein.

### Cellular ATP levels

The amount of intracellular ATP was determined with the CellTiter-Glo^®^ Luminescent Cell Viability Assay (Promega AG, Switzerland). SH-SY5Y cells were seeded in a 96-well plate at a density of 3.5 × 10^4^ cells and cultured as described above. The measurements were performed according to the supplier’s datasheet. Shortly, the CellTiter-Glo substrate was reconstituted in the CellTiter-Glo buffer and equilibrated to room temperature. Prior to the measurement (30 min) the plate was equilibrated to room temperature. The staining solution was added, mixed with an orbital shaker for 2 min and incubated in the dark for 10 min to stabilize the signal. Following, the luminescence was measured with a Synergy H1 multi-mode reader with an integration time of 1 s. Finally, the protein amount of each sample was determined with OPA as described before. The values were normalized to 1 mg protein.

### Superoxide measurements

Mitochondrial superoxide was measured with the MitoSOX™ Red mitochondrial superoxide indicator for live cell imagining (Molecular Probes, Thermo Fisher Scientific, Switzerland). SH-SY5Y cells were seeded in a 96-well plate (Huberlab, Switzerland) at a density of 3.5 × 10^4^ cells and cultured as described above. The measurements were done according to the supplier’s datasheet. Briefly, a [5 mM] stock solution of the provided MitoSOX reagent was prepared in DMSO and the stock solution was further diluted to a [5 µM] working solution in Hank’s balanced salt solution with calcium and magnesium (HBSS, Sigma, Switzerland). At DIV8, non-differentiated, differentiated as well as nanoparticle-treated SH-SY5Y cells were incubated with [5 µM] MitoSOX working solution at 37 °C for 50 min after the cells were washed with HBSS. Fluorescence measurements were performed immediately at an emission of 510 nm and excitation of 580 nm with a Synergy H1 multi-mode reader (BioTek, Switzerland). Subsequently, the protein amount of each sample was determined using Fluoraldehyde™ o-phthaldialdehyde reagent solution (OPA, Thermo Fisher Scientific, Switzerland) at an emission of 360 nm and an excitation of 460 nm. The results of the MitoSOX Assay were normalized to 1 mg protein.

### Protein expression of differentiation markers and OXPHOS enzymes

SH-SY5Y cells were cultured as described above. On DIV7, protein extraction was performed and the samples were analyzed by Western Blot as described previously [5]. Briefly, SH-SY5Y cells were lysed in protein lysis buffer containing phosphatase (Sigma, Switzerland) and protease (Thermo ScientificTM, IL, Switzerland) cocktail inhibitors. The lysed cell suspension was incubated on ice for 15 min, sonicated for 10 s and finally centrifuged at high speed for 10 min at 4 °C. The protein content of the supernatant was quantified using the Pierce protein assay reagent (Bio-Rad Laboratories, CA, USA); 10 µg of protein from each test sample were loaded and subsequently separated on 12% SDS-PAGE before the protein was transferred onto PVDF membrane (Sigma, USA) at 0.25 A for 1.5 h (PI3- and ERK-kinases, OXPHOS) or nitrocellulose membrane (Bio-Rad, Switzerland) at 0.35 A for 2 h (MAP-2) membranes. Blocking for 2 h in PBS 0.2% Tween, 5% milk was performed at room temperature. Subsequently, the primary antibodies, rabbit anti-phospho-Akt (1:1000), rabbit anti-Akt (1:1000), mouse anti-phospho-pMAP-K (ERK1/2) (1:2000), mouse anti-pMAP-K (ERK1/2) (1:2000) all from Cell Signaling Technology (MA, USA); mouse anti-MAP-2 (1:500) and the monoclonal mouse antibody anti-β-actin (1:10,000) both from Sigma (MO, USA); mouse anti-OXPHOS cocktail (1:1000) from Abcam (UK) were added for overnight incubation at 4 °C. The secondary antibodies, donkey-anti-rabbit and donkey-anti-mouse horseradish peroxidase (Thermo ScientificTM, IL, USA) were applied accordingly at 1:10,000–1:20,000 for 2 h at room temperature. Chemiluminescent substrate (Advansta Western Bright Sirius Chemiluminescent, Witec, Switzerland) was used and chemiluminescence was detected using a luminescent image analyzer (LAS-3000 Imaging System from Fuji, Japan). Blot quantification was performed using ImageJ analysis (NIH, Bethesda, USA) measuring the ratio between the intensity of the obtained band of a specific marker versus the corresponding band of actin using arbitrary units.

### Neuronal differentiation

Immunofluorescence staining was performed at the end of the culture period as previously described [5]. Briefly, cultures were fixed with 4% paraformaldehyde, blocking was performed with 10% normal horse serum in 0.4% Triton-X PBS before the primary antibody mouse anti-β-3-tubulin (1:500) (Sigma, Switzerland) was applied overnight at 4 °C in 0.4% Triton-X PBS. Finally, the secondary antibody [Alexa Fluor donkey anti-mouse 488 nm, Alexa Fluor anti-goat 488 nm (1:250) (Molecular Probes, Thermo Fisher Scientific, Switzerland)] was applied. Cell nuclei were counterstained using Hoechst 333,342 (1:10,000) (Molecular Probes, Thermo Fisher Scientific, Switzerland). Images were performed using a Zeiss Axio Imager Z1 coupled with an Apotome 1 (Carl Zeiss Vision Swiss AG, Feldbach, Switzerland).

### Statistical analysis

Three to five independent experiments were performed for all parameters measured. Data at each stage were analyzed using a one-way ANOVA followed by Tukey’s multiple comparison to compare the means of all treatments with the respective controls (GraphPad Software Inc., La Jolla, USA). For the protein quantification, three to four independent experiments were performed in duplicates for all analyses. A one-way ANOVA followed by the Dunnett’s test was used for group comparisons. Data are presented as mean ± standard error of the mean (SEM). p values ≤0.05 were considered significant.

## Results

### Oxygen consumption rate and extracellular acidification rate

**Fig. 1:**
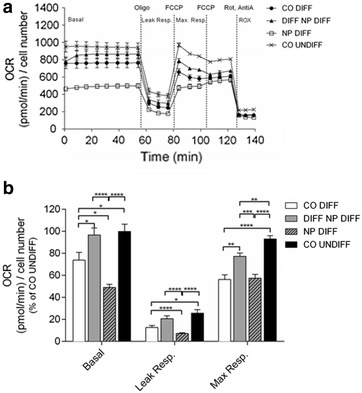
The oxygen consumption rate (OCR) was measured using a Seahorse Biosciences XF24 Analyser in SH-SY5Y neuroblastoma cells exposed to PCL-NPs for 24 h before differentiation (NP DIFF) or during differentiation (DIFF NP DIFF) on day in vitro (DIV) 1 and DIV4, respectively. Untreated SH-SY5Y cells, either non-differentiated (CO UNDIFF) or differentiated for 6 days (CO DIFF), were analyzed in parallel (**a**, **b**). SH-SY5Y cells were exposed sequentially to each mitochondrial modulator of mitochondrial activity (oligomycin = Oligo, FCCP and rotenone/antimycin A = Rot, AntiA). The mean ± SEM of the mitochondrial stress test on OCR are depicted over time (**a**). A quantitative analysis of the data is depicted in (**b**). Values represent the mean + SEM; n = 5 replicates of five independent experiments. *p ≤ 0.05; **p ≤ 0.01; ***p ≤ 0.001; ****p ≤ 0.0001

To evaluate whether PCL-NP exposure before and during differentiation affected mitochondrial function, the oxygen consumption rate (OCR) and the extracellular acidification rate (ECAR) were analyzed in differentiated and undifferentiated SH-SY5Y cells. As shown in Fig. 1, the differentiation process led to a significant reduction (26.2%, p ≤ 0.05) of basal OCR and a significant reduction (36.8%, p ≤ 0.0001) of maximal respiration in SH-SY5Y cells when compared to undifferentiated control. PCL-NP exposure starting before differentiation was associated with a significantly decreased basal OCR compared to undifferentiated controls as well as differentiated controls (50.9 and 24.7%, respectively) as shown in Fig. 1b. Leak respiration was decreased compared to undifferentiated controls and differentiated controls, whereas maximal respiration was approximately the same as in differentiated control cells, but lower compared to undifferentiated control cells.

The PCL-NP exposure initiated on the third day of differentiation resulted in a non-significant decrease (3.25%) in OCR as compared to undifferentiated controls, but a significant increase of 22.9% when compared to differentiated controls (p ≤ 0.05) (Fig. 1b). Leak respiration and maximal respiratory capacity were significantly lower compared to undifferentiated controls, but significantly higher compared to differentiated control cells (Fig. 1b).

The maximal respiratory capacity for SH-SY5Y cells exposed to PCL-NPs started during differentiation was significantly lower compared to undifferentiated controls (p ≤ 0.01), but significantly higher compared to differentiated control cells (p ≤ 0.01) (Fig. 1b).

**Fig. 2:**
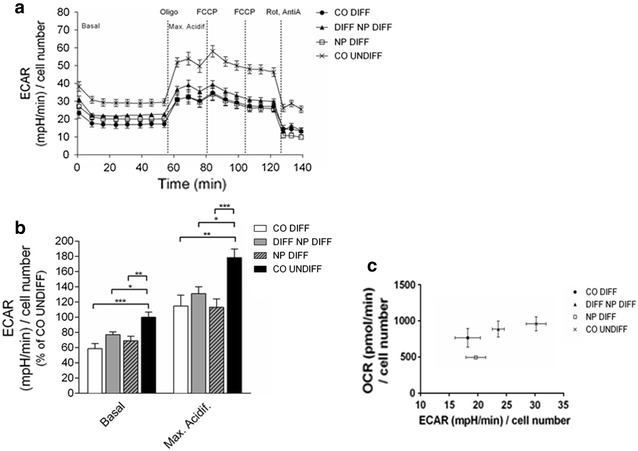
The extracellular acidification rate (ECAR) was measured using a Seahorse Biosciences XF24 Analyser. The bioenergetic activity was analyzed in SH-SY5Y neuroblastoma cells exposed to PCL-NPs for 24 h before differentiation (NP DIFF) or during differentiation (DIFF NP DIFF) on day in vitro (DIV) 1 and DIV4, respectively. Untreated SH-SY5Y cells, either non-differentiated (CO UNDIFF) or differentiated for 6 days (CO DIFF), were analyzed in parallel (**a**, **b**). SH-SY5Y cells were exposed sequentially to each mitochondrial modulator of mitochondrial activity (oligomycin, FCCP and rotenone/actinomycin A). The mean ± SEM of the mitochondrial stress test on ECAR are depicted over time (**a**). A quantitative analysis of the data is depicted in (**b**). Values represent the mean + SEM; n = 5 replicates of five independent experiments. *p ≤ 0.05; **p ≤ 0.01; ***p ≤ 0.001; ****p ≤ 0.0001. The energy phenotype is depicted by ECAR on the *x* axis and the mitochondrial respiration is represented by the oxygen consumption rate (OCR) on the *y* axis. Bioenergetic profiling of SH-SY5Y cells (OCR versus ECAR) revealed decreased metabolic activity after exposure to PCL-NPs. Values represent the raw mean ± SEM of each group (mean of the ECAR in abscissa/mean of the OCR in ordinate) (**c**)

Under basal conditions, the ECAR was significantly decreased (41.4%) in differentiated cells compared to undifferentiated cells (p ≤ 0.001) as illustrated in Fig. 2a, b. The ECAR in cells exposed to PCL-NPs before and during differentiation was significantly lower compared to undifferentiated SH-SY5Y control cells with p ≤ 0.01 and p ≤ 0.05, respectively (Fig. 2b). No difference was found between differentiated SH-SY5Y cells devoid of nanoparticles and PCL-NP exposed cells.

The inhibition of ATP synthesis initiated by oligomycin resulted in an increase in ECAR in all four groups due to the inhibition of mitochondrial ATPase with a similar pattern for maximal acidification. The increase was 1.8-fold in undifferentiated cells and twofold in differentiated cells (p ≤ 0.01) as shown in Fig. 2.

The cellular metabolic phenotypes are summarized in Fig. 2c. Whereas undifferentiated SH-SY5Y cells are characterized by a high cellular OCR and ECAR, differentiation process leads to a shift to lower OCR and ECAR levels. Exposures to nanoparticles before and during neuronal differentiation induce significant changes in the described shift in energy metabolism (OCR and ECAR).

### Expression and activity of mitochondrial respiratory chain enzymes

**Fig. 3:**
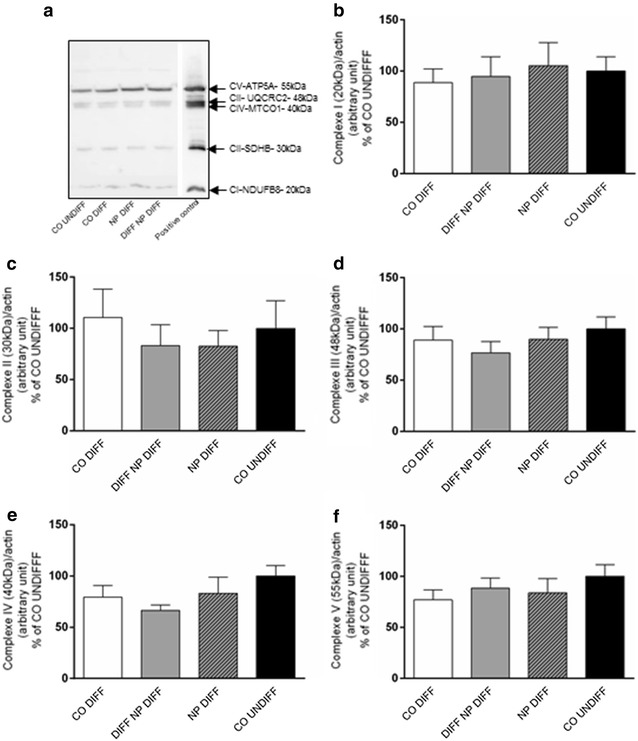
Oxidative phosphorylation enzymes (OXPHOS) were analyzed to evaluate expression of mitochondrial chain complexes I to V in SH-SY5Y cells exposed to PCL-NPs ([2.6 × 10^10^ PCL-NPs/ml]) for 24 h before (DIV1, NP DIFF) or after (DIV4, DIFF NP DIFF) differentiation with retinoic acid. Untreated controls, undifferentiated SH-SY5Y cells (CO UNDIFF) and differentiated SH-SY5Y cells (CO DIFF) were performed in parallel. The following complexes or subunits of the complexes were analyzed: Accessory subunit of the mitochondrial membrane respiratory chain NADH dehydrogenase complex (C)I (CI-NDUFB8); iron-sulfur protein (IP) subunit of succinate dehydrogenase involved in CII (CII-SDHB); component of the ubiquinol-cytochrome c reductase of CIII (CIII-UQCRC2); catalytic subunit of cytochrome c oxidase of CIV (CIV- MTCO1), and mitochondrial membrane ATP synthase (F(1)F(0) ATP synthase) or CV (CV-ATP5A). Representative Western blots are shown in (**a**). The histograms represent the ratio of each complex normalized to the corresponding actin (loading control) band CI (**b**), CII (**c**), CIII (**d**), CIV (**e**) and CV (**f**). Respective protein levels were assessed using digital quantification of immunoblots, and they are presented as relative intensity compared to total protein. The analysis was done using ImageJ. Values are expressed with arbitrary units. Error bars represent the mean + SEM

In order to gain more information about the mechanism of the observed decrease in OCR measurements, Western blot analyses of subunits of each enzyme complex were performed and enzymatic activities of all five respiratory chain complexes were quantified. As shown in Fig. 3, the protein content of selected subunits of enzyme complexes I to V of the respiratory chain revealed no significant change after PCL-NP exposure when compared to undifferentiated or differentiated control cells. In accordance, activity measurements of complexes I–V showed no significant variation (Table 1) independent of the cell differentiation and the nanoparticle exposure time.

**Table 1:** Enzymatic activities of respiratory chain complexes

Experimental group	Citrate synthase activity (mU/mg protein)	Enzymatic complex activities (mU/mU citrate synthase)				
		CI	CII	CIII	CIV	CV
CO DIFF	111 ± 7	0.1 ± 0.01	0.15 ± 0.01	0.13 ± 0.01	0.18 ± 0.01	0.39 ± 0.03
DIFF NP DIFF	131 ± 8	0.07 ± 0.01	0.14 ± 0.01	0.14 ± 0.01	0.13 ± 0.01	0.35 ± 0.03
NP DIFF	127 ± 9	0.08 ± 0.01	0.16 ± 0.02	0.14 ± 0.01	0.15 ± 0.01	0.37 ± 0.05
CO UNDIFF	118 ± 12	0.08 ± 0.01	0.14 ± 0.01	0.18 ± 0.02	0.13 ± 0.03	0.40 ± 0.04

Enzymatic activities of respiratory chain complexes were measured on day in vitro 8 (DIV8) in undifferentiated control cells (CO UNDIFF), differentiated control cells (CO DIFF) and PCL-NPs exposed cells before (NP DIFF) or during (DIFF NP DIFF) differentiation. Values were estimated by the difference in activity levels measured in presence and absence of specific inhibitors; values are expressed as ratios to the mitochondrial marker enzyme citrate synthase (mU/mU citrate synthase) ± S.E.M

### Phosphofructokinase activity

**Fig. 4:**
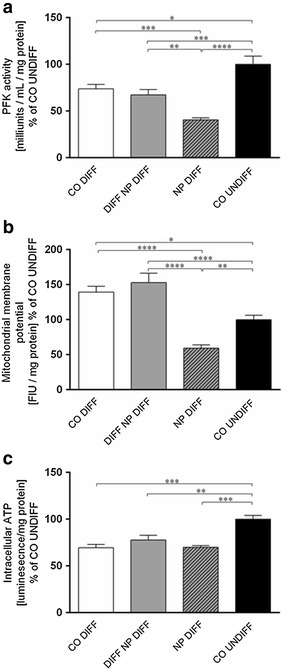
Phosphofructokinase (PFK) activity (**a**), mitochondrial membrane potential (ΔΨ_m_) (**b**) and ATP production (**c**) were assessed in SH-SY5Y cells undifferentiated controls (CO UNDIFF), differentiated controls (CO DIFF) and exposed to PCL-NPs before (NP DIFF) or during (DIFF NP DIFF) differentiation. The histograms represent values calculated as percent of undifferentiated controls. Values are expressed as milliunits/ml/mg protein for PKF activity (**a**), fluorescence intensity unit (FIU)/mg protein for ΔΨ_m_ (**b**), as luminescence/mg protein for ATP production (**c**). Error bars represent the mean + SEM. Significant differences to undifferentiated and differentiated controls are labeled with *asterisks* (*p ≤ 0.05; **p ≤ 0.01; ***p ≤ 0.001; ***p ≤ 0.0001)

Phosphofructokinase (PFK) activity in SH-SY5Y cells was found significantly decreased in differentiated controls in comparison to undifferentiated controls (p ≤ 0.05) (Fig. 4a). In contrast to nanoparticle exposure during differentiation, nanoparticle exposure before differentiation, led to a significant decrease in PFK activity when compared to differentiated controls (p ≤ 0.0001). PFK activity was also found to be significantly reduced in nanoparticle treated cells compared to undifferentiated control cells with the effect being more pronounced for nanoparticles applied before differentiation (Fig. 4a).

### Mitochondrial membrane potential (ΔΨ_m_), intracellular ATP and superoxide production

Mitochondrial membrane potential is a key indicator of the integrity of the membrane. As shown in Fig. 4b, differentiation of SH-SY5Y cells led to a significant increase in ΔΨ_m_ compared to undifferentiated control cells (p ≤ 0.05) as well as nanoparticle exposure during differentiation. In contrast, exposure to PCL-NPs before differentiation resulted in a significant reduction in ΔΨ_m_ when compared to differentiated controls (p ≤ 0.0001) and to undifferentiated controls (p ≤ 0.01) (Fig. 4b).

As illustrated in Fig. 4c, significant differences in ATP production on DIV8 were observed between undifferentiated controls and differentiated controls (p ≤ 0.001) and cells exposed to nanoparticles before differentiation (p ≤ 0.01) and during differentiation (p ≤ 0.001).

**Fig. 5:**
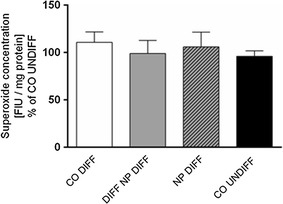
Superoxide production was measured at DIV8 in SH-SY5Y cells exposed to PCL-NPs before (DIV1, NP DIFF) or during (DIV4, DIFF NP DIFF) differentiation with RA. Untreated controls, undifferentiated cells (CO UNDIFF) and differentiated cells (CO DIFF) were performed in parallel. Values are expressed as fluorescent intensity units (FIU)/mg protein; they are represented as percentage of undifferentiated control. Error bars represent the mean + SEM

No significant variation in superoxide production was found when PCL-NP exposed groups and controls were compared (Fig. 5).

### Effect of NP exposure on neuronal differentiation markers

**Fig. 6:**
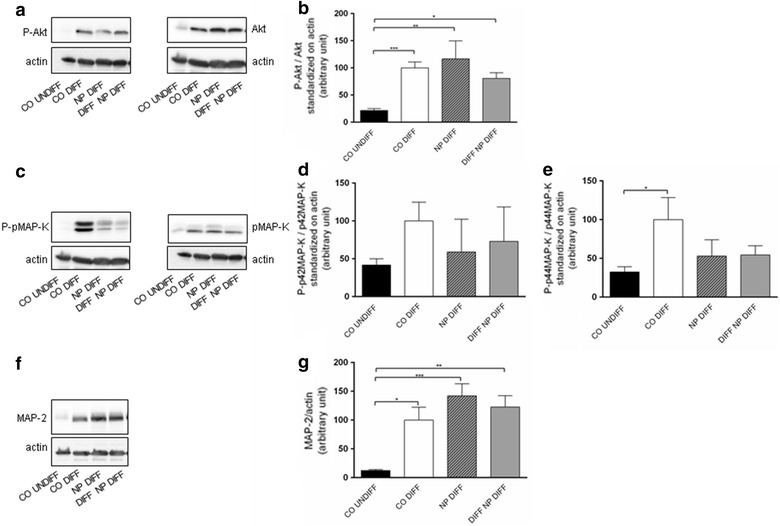
Markers of the PI3 kinase pathways (PI3-K/Akt), MAP kinase (MAP-K/ERK), and of mature neurons (MAP-2) were used to evaluate neuronal differentiation in SH-SY5Y cells exposed to PCL-NPs ([2.6 × 10^10^] PCL-NPs/ml) for 24 h before (DIV1, NP DIFF) or during (DIV4, DIFF NP DIFF) differentiation with retinoic acid added until DIV7. Untreated controls, undifferentiated SH-SY5Y cells (CO UNDIFF) and differentiated SH-SY5Y cells (CO DIFF) were performed in parallel. Representative Western blots are shown in (**a**, **c**, **f**). The histograms represent the ratio of phosphorylated Akt (P-Akt) (**b**) and phosphorylated p42/44-MAP-K (P-p42/44-MAP-K) (**d**, **e**) to Akt and p42/44-MAP-K. A histogram of the ratio MAP-2/actin is shown in (**g**). The actin signal was used as loading control. Respective protein levels were assessed using digital quantification of immunoblots, and they are presented as relative intensity compared to total protein. The analysis was done using ImageJ. Values are expressed with arbitrary units. Error bars represent the mean + SEM. Significant differences to undifferentiated and differentiated controls are labeled with asterisks (*p ≤ 0.05; **p ≤ 0.01; ***p ≤ 0.001)

Kinases have been demonstrated to be involved in neurite elongation (PI3-K/Akt) and in synaptic plasticity (MAP-K/ERK) of neurons. Therefore, we investigated the effect of PCL-NP exposure on the expression of these kinases. A significant upregulation of the phosphorylated kinases Akt (P-Akt) (p ≤ 0.001) and MAP-K (P-p42/44-MAP-K) (p ≤ 0.05) was found in differentiated cells compared to undifferentiated SH-SY5Y cells as shown in Fig. 6a–e. PCL-NP exposure for 24 h before differentiation and during differentiation resulted in a significant upregulation of phosphorylated-Akt (P-Akt) (p ≤ 0.01) of the same magnitude when compared to undifferentiated control SH-SY5Y cells on DIV6 (Fig. 6b). No difference between PCL-NP treated cells and differentiated control cells was found. Likewise, a decrease in both, P-p42-MAP-K and P-p44-MAP-K was seen in both PCL-NP exposed conditions in SH-SY5Y cells when compared to differentiated control cells. However, this effect was not statistically significant (Fig. 6d, e).

Differentiated SH-SY5Y cells showed a significant increase in the differentiation marker MAP-2 (p ≤ 0.05) (Fig. 6f, g). Exposure to PCL-NPs before and during the differentiation resulted in a significant upregulation of this marker when compared to undifferentiated control cells (p ≤ 0.001 and p ≤ 0.01, respectively) as illustrated in Fig. 6g. The upregulation of MAP-2 after PCL-NP exposure was higher compared to differentiated controls, but the effect was not statistically significant.

**Fig. 7:**
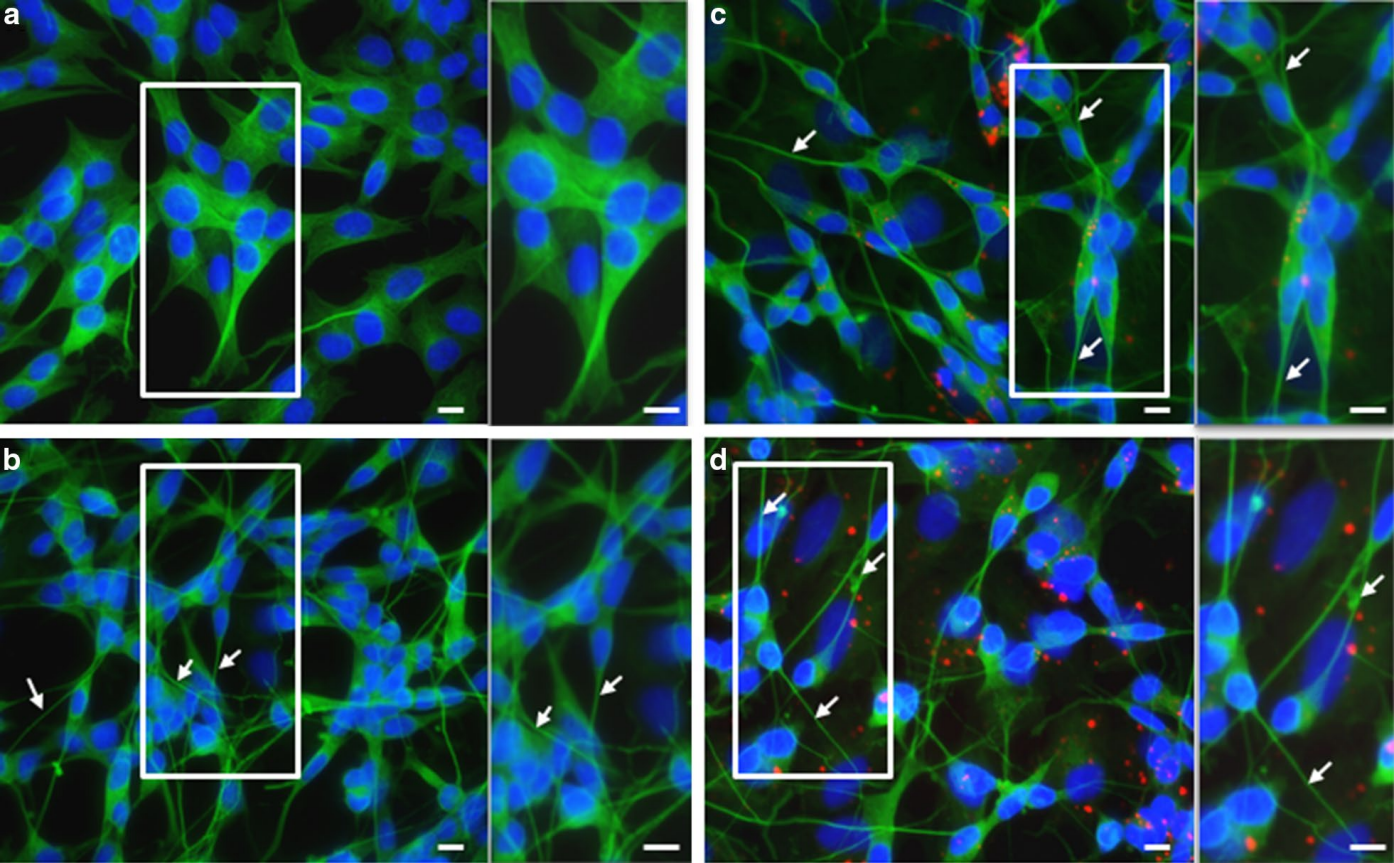
Representative microscopic images showing morphological features of SH-SY5Y cells, undifferentiated controls (**a**), differentiated controls (**b**) and exposed to NPs before (**c**) or during differentiation (**d**). At the end of the differentiation (DIV8), cells were stained with the neuronal marker β-3-tubulin (*green*), NPs appear in *red* (RITC labeling) and cell nuclei were counterstained with Hoechst (*blue*). The arrows depict neurites from differentiated neurons. Magnification 400×. Inserts demonstrate higher magnification of the *square* in the picture. *Scale bars* 10 μm

Neuronal differentiation was analyzed using β-3-tubulin-staining and differentiated cells displayed typical neuronal morphology (Fig. 7). Neurite outgrowth was observed when undifferentiated control cells (Fig. 7a) and differentiated controls (Fig. 7b) were compared. Nanoparticle exposure (Fig. 7c, d) did not result in obvious cellular morphological differences when compared to differentiated control cells.

## Discussion

A limited glycolytic potential has been linked to neurodegeneration and mitochondrial dynamics are important for neurodegeneration [9]. In contrast to astrocytes, neurons were reported to exhibit lower levels of glycolysis [26].

Differentiation of SH-SY5Y cells resulted in changes in the cellular metabolism. As expected, the OCR and the intracellular ATP levels were decreased in differentiated SH-SY5Y cells compared to undifferentiated cells corroborating previous findings [27, 28]. Differentiated SH-SY5Y cells were reported to exhibit an elevated stimulation of mitochondrial respiration indicating an increased mitochondrial reserve capacity compared to undifferentiated SH-SY5Y cells due to changes in mitochondrial metabolism [29, 30].

The basal OCR was significantly reduced after PCL-NP exposure before differentiation compared to differentiated cells, whereas an increase in OCR was measured when PCL-NPs were added to the cells during differentiation. Blockage or serious inhibition of the mitochondrial chain is detrimental for neuronal differentiation [31, 32]. ZnO nanoparticles were reported to induce apoptosis and decrease the mitochondrial membrane potential in primary astrocytes indicating that mitochondria are involved in ZnO nanoparticle-induced apoptosis. The nanoparticle exposure resulted in phosphorylation of c-Jun *N*-terminal kinase (JNK), ERK, and p38 mitogen activated protein kinase (p38 MAP-K) [14]. Silver nanoparticles showed a decoupling effect on mitochondria resulting in an impairment of mitochondrial oxidative phosphorylation [16]. In agreement with the observed increase in basal cellular respiration in PCL-NPs exposure during differentiation, TiO_2_ nanoparticles increased the basal cellular respiration in human keratinocytes in a concentration-dependent manner [33].

In undifferentiated and differentiated SH-SY5Y control cells, a decrease in OCR occurred after the addition of the inhibitor of ATP synthase, oligomycin, with the drop being more pronounced in differentiated controls indicating that the mitochondrial oxygen consumption used for ATP synthesis increases during cellular differentiation. The observed increase in mitochondrial membrane potential in differentiated SH-SY5Y cells compared to undifferentiated cells has been described before [29]. The increase in membrane potential was also found after nanoparticle exposure during differentiation, whereas nanoparticle exposure before differentiation resulted in a significant reduction of mitochondrial membrane potential indicating an impairment of mitochondrial metabolism. As the elevation in membrane potential has been demonstrated to be accompanied with differentiation [29], the nanoparticle exposure might impair differentiation. Silica-oxide nanoparticles were reported to reduce the membrane potential in hepatocytes [34], and a decrease in membrane potential was also demonstrated in human umbilical endothelial cells after exposure to 60 nm silica nanoparticles [35] corroborating our findings. Production of ATP by oxidative phosphorylation and especially dysfunction of this system has been related to the generation of superoxide, and its scavenging is accomplished by radical scavengers such as superoxide dismutase [6]. PCL-NPs exposure before differentiation decreased basal OCR significantly more compared to differentiated control cells. In contrast, PCL-NP exposure during differentiation did not significantly change ATP production compared to differentiated control cells. Additional measurements showed that differentiation induced a significant decrease in ATP levels, but nanoparticle exposure had no effect on this parameter. In addition, the expression and the activity of the mitochondrial chain complexes I to V was not affected by nanoparticle exposure. In contrast, silica oxide nanoparticles were demonstrated to reduce the mitochondrial chain complexes I, III and IV in rat hepatocytes [34]. The smaller size of these nanoparticles as well as the different cell type may explain the different findings. Acute nanoparticle exposure might increase leak respiration if given to undifferentiated cells causing acute stress. Hence, they are more vulnerable to mitochondrial uncoupling. In agreement with this hypothesis, transient ROS production was found in undifferentiated SH-SY5Y cells [3]. Superoxide production did not vary between all groups in this study. Previously, an increase in ROS production was found in undifferentiated cells [3]. Silica nanoparticles were also reported to increase superoxide levels in the corpus striatum of rats [36] and inhibited superoxide dismutase in human endothelial cells [35]. However, the physicochemical differences and/or the different species or cell type may explain the variable findings. It needs to be noted that distinct energy metabolism profiles were found in pluripotent stem cells, differentiated cells and cancer cells [37]. Recently, it was demonstrated that changes in mitochondrial function after Fe_3_O_4_ nanoparticle exposure were less pronounced in neuronal cells compared to astrocytes [38]. In the present study, cells were differentiated and the superoxide was measured after the differentiation period. Hence, the discrepancy may be due to the differentiation of the cells and the time of the measurements. The production of oxygen radicals by HeLa cells and human hepatocytes after silica-quantum dots exposure was reported to reach a plateau already 40 min after exposure [39]. An increase in superoxide cannot be excluded in our study as it might have occurred right after the nanoparticle exposure or before the end of the differentiation period.

The uncoupled maximal respiration (OCR under FCCP) was significantly higher in undifferentiated cells compared to differentiated cells. PCL-NP exposure before differentiation did not change maximal respiration, whereas PCL-NP exposure during differentiation significantly increased maximal respiration compared to differentiated control cells. TiO_2_ nanoparticle exposure was demonstrated to change mitochondrial membrane potential in rat primary hippocampal neurons [40], induce neuronal dysfunction in primary astrocytes [41], and decrease the activity of all mitochondrial respiratory chain complexes in brain tissue [42]. These findings corroborate our data despite the different composition of nanoparticles. In contrast, TiO_2_ nanoparticles were reported to increase the OCR after rotenone in keratinocytes [33]. Different material, size and charge of the nanoparticles and the difference in cell origin may account for this difference.

Mitochondria play an important role in cell metabolism during neuronal differentiation because neuronal cell differentiation requires metabolic adaptations [9]. A lower glycolytic activity was found in differentiated cells compared to undifferentiated cells, and PCL-NP exposure, independent of the time of exposure, was not significantly different from differentiated cells but significantly reduced compared to undifferentiated controls. Carbon monoxide supplementation was demonstrated to promote metabolic changes occurring during neuronal differentiation, namely, from glycolytic to oxidative metabolism [9]. Measurements of the activity of the key enzyme in glycolysis, PFK, showed a significant reduction between differentiated control cells and undifferentiated cells. PFK levels of cells exposed to PCL-NPs during differentiation did not vary compared to differentiated controls, but PFK levels were significantly reduced in cells where nanoparticles were given before differentiation. Cell differentiation was reported to be associated with metabolic changes and a metabolic shift from glycolysis to oxidative phosphorylation during neuronal differentiation has been demonstrated [19]. Moreover, artificial constitutive expression of hexokinase and lactate dehydrogenase was shown to result in cell death indicating that a decrease in glycolysis is essential for neuronal differentiation [43]. Thus the observed reduction of PFK activity in cells exposed to PCL-NPs before differentiation may affect differentiation. In contrary, an increase in glucose metabolism was found during neuronal differentiation with PI3-K/Akt/mTOR signaling being a critical regulator [14].

## Neuronal differentiation

Kinases such as PI3-K/Akt and MAP-K/ERK are involved in neurite elongation, neuronal survival and synaptic plasticity of neurons [10, 21–23], and MAP-K/ERK was demonstrated to play a key role in mitochondrial function [44]. Recent data demonstrate that proliferative neuronal stem cells have high ROS levels, which is required for self-renewal and neurogenesis with underlying PI3-K/Akt signaling [45]. A significant increase in the differentiation markers (PI3-K/Akt and MAP-K/ERK) was found in differentiated SH-SY5Y compared to undifferentiated cells indicating that these kinases are involved in neuronal differentiation [46]. This finding is corroborating previously published data [5] and other studies also demonstrated that the PI3-K/Akt improved neurite elongation in primary hippocampal and cortical neurons [21, 22, 47]. However, PCL-NP exposure before and during differentiation did not significantly change the expression of both kinases. In contrast, recently published results demonstrated a significant reduction of the differentiation markers used in this study, but neurite outgrowth was not significantly altered by exposure to PCL-NPs for 24 h before differentiation in SH-SY5Y cells [5]. The disparate findings may be explained by the differentiation protocol used in the current study as compared to the previous work; namely, a moderate FBS starvation during RA treatment. Starvation was only initiated after DIV3 (1% FBS) and the cells were incubated with 5% FBS for the first 3 days of differentiation. The expression of the neuronal marker MAP-2 was significantly increased in differentiated control cells and in cells exposed to PCL-NPs either before or during differentiation. Previously published data demonstrated a significantly reduced expression of MAP-2 in SH-SY5Y cells exposed to PCL-NP for 24 h before differentiation supporting this hypothesis. However, the trend towards a reduced expression of PI3-K/Akt and MAP-K/ERK was also found in the present study. In contrast to these findings, an activation of the kinases Akt and ERK after exposure to silver nanoparticles, enhanced neurite outgrowth, an increase in MAP-2 and an increased ROS production was reported [23]. ERK induction was found to be involved in mitochondrial degradation in SH-SY5Y cells used in an in vitro model for Parkinson disease with its activity being important for microphagy [48, 49].

The size (30 vs. 80 nm) and the different material may explain the disparate findings. Silica nanoparticles were shown to cause oxidative stress in endothelial cells via activation of the MAP-K/Nrf2 pathway and nuclear factor-kappaB signaling [50]. The same authors reported that amorphous silica nanoparticles induced ROS production mediated by MAP-K/Bcl-2 and PI3-K/Akt/mTOR signaling in endothelial cells. In that study, phosphorylated ERK, PI3-K/Akt, and mTOR were significantly decreased, whereas phosphorylated JNK and p38 MAP-K were increased after exposure to silica nanoparticles [51]. Despite the different cell origin, in our study, a decreased PI3-K/Akt and phosphorylated p42/44-MAP-K was found after PCL-NP exposure before and during differentiation in SH-SY5Y cells even though the effect was not statistically significant. As reported previously, PCL-NP exposure increased ROS production only transiently in SH-SY5Y cells [3]. Oxidative stress was demonstrated to activate MAP-K pathways [20]. Silver nanoparticles were shown to result in an increase in ROS and an activation of ERK and Akt supporting neuronal differentiation in SH-SY5Y cells. This was also demonstrated by an increased expression of the neuronal differentiation marker MAP-2 [23]. This finding is in agreement with our data, where PCL-NP exposure led to slightly increased levels of MAP-2.

## Conclusions

PCL-NP exposure of SH-SY5Y cells affected mitochondrial function and the expression of differentiation markers in a time-dependent manner during differentiation. Considering the importance of adaptations in cellular respiration for neuronal differentiation and function, further studies addressing the regulation and the functional impact of PCL-NP exposure are needed to unravel the underlying mechanisms and consequences to assess the possible risks for using them in biomedical applications as impaired mitochondrial function may lead to neurodegeneration.

## Abbreviations

ATP: adenosine triphosphate
C I-V: complexes I to V
DIV: day in vitro
DMEM: Dulbecco’s Modified Eagle Medium
FBS: fetal bovine serum
ECAR: extracellular acidification rate
HBSS: Hank’s balanced salt solution with calcium and magnesium
ICG: idocyanine green
MAP-2: microtubule associated protein-2
MAP-K/ERK: mitogen activated protein kinase/extracellular signal-related kinases
ΔΨ_m_: mitochondrial membrane potential
O_2_: oxygen
OCR: oxygen consumption rate
OPA: Fluoraldehyde™ o-phthaldialdehyde reagent solution
PCL-NPs: silica-indocyanine green/poly-(ε-caprolactone) nanoparticles
PFK: phosphofructokinase
PI3-K/Akt/mTOR: phosphatidyl-inositol 3-kinase/serine/threonine specific protein kinase/mechanistic target of rapamycin
RA: retinoic acid
ROS: reactive oxygen species
SEM: standard error of the mean
